# Right hepatectomy under cardiopulmonary bypass for hepatocellular carcinoma with inferior vena cava tumor thrombus: a case report

**DOI:** 10.1186/s40792-023-01756-y

**Published:** 2023-10-03

**Authors:** Hiroki Kushiya, Yoshiyasu Ambo, Minoru Takada, Takahiko Masuda, Shuichi Naraoka, Satoshi Hirano

**Affiliations:** 1 Teine Keijinkai General Hospital, maeda 1 jo 12 Choume, Teine-ku, Sapporo, 006-0811 Japan; 2 Department of Gastroenterological Surgery II, Hokkaido University Faculty of Medicine, Kita 15 Nishi 7, Kita-ku, Sapporo, 060-8638 Japan

**Keywords:** Hepatocellular carcinoma, Tumor thrombus, Cardiopulmonary bypass

## Abstract

**Background:**

The prognosis of hepatocellular carcinoma (HCC) with vascular invasion is extremely poor, especially in patients with tumor thrombus (TT) of the inferior vena cava (IVC), which is an oncological emergency with a high risk of sudden death due to TT extension or migration. Herein, we describe a case of HCC with TT of the IVC that rapidly extended into the right atrium (RA), in which right hepatectomy was performed under cardiopulmonary bypass.

**Case presentation:**

A 64-year-old man was diagnosed with HCC with IVC TT, and right hepatic lobectomy was scheduled. While awaiting surgery, he complained of respiratory distress and rushed to the emergency room. The TT had reached the RA, and the patient was in a state of oncologic emergency. We requested the cooperation of the cardiovascular surgery department, and under artificial cardiopulmonary support, the right atrium was incised, and a part of the TT was removed. The IVC was clamped to prevent tumor dispersal, and right hepatic lobectomy was performed. The remaining thrombus was excised along with the right lobe of the liver by incising the IVC. There were no serious postoperative complications, and the patient is alive 1 year and 5 months postoperatively.

**Conclusion:**

Hepatic resection with cardiopulmonary bypass could be an option for HCC with TT reaching the RA.

## Introduction

The effectiveness of liver resection for hepatocellular carcinoma (HCC) with hepatic vein tumor thrombus (HVTT) has been previously reported [1]. However, the prognosis of HCC with tumor thrombus (TT) in the inferior vena cava (IVC) or right atrium (RA) is extremely poor due to systemic metastasis and sudden death from pulmonary embolism or occlusion of the tricuspid valve [2, 3]. Additionally, because of the rarity of HCC with TT in the IVC, optimal treatment strategies are not clear. Herein, we present a case of right hepatectomy performed under cardiopulmonary bypass for HCC with TT in IVC.

## Case presentation

**Fig. 1:**
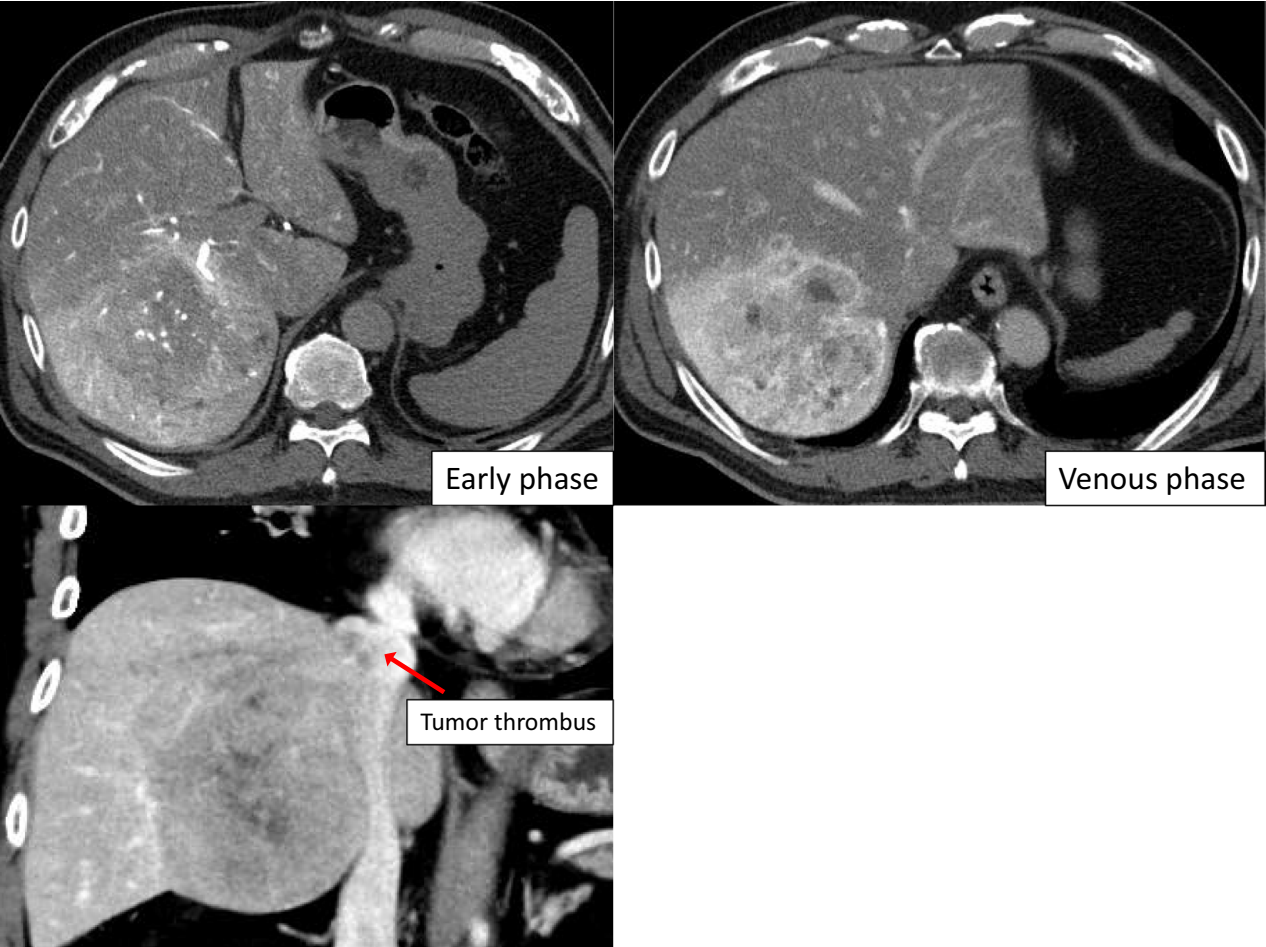
Abdominal computed tomography showed a tumor with tumor thrombus extending to the IVC

**Fig. 2:**
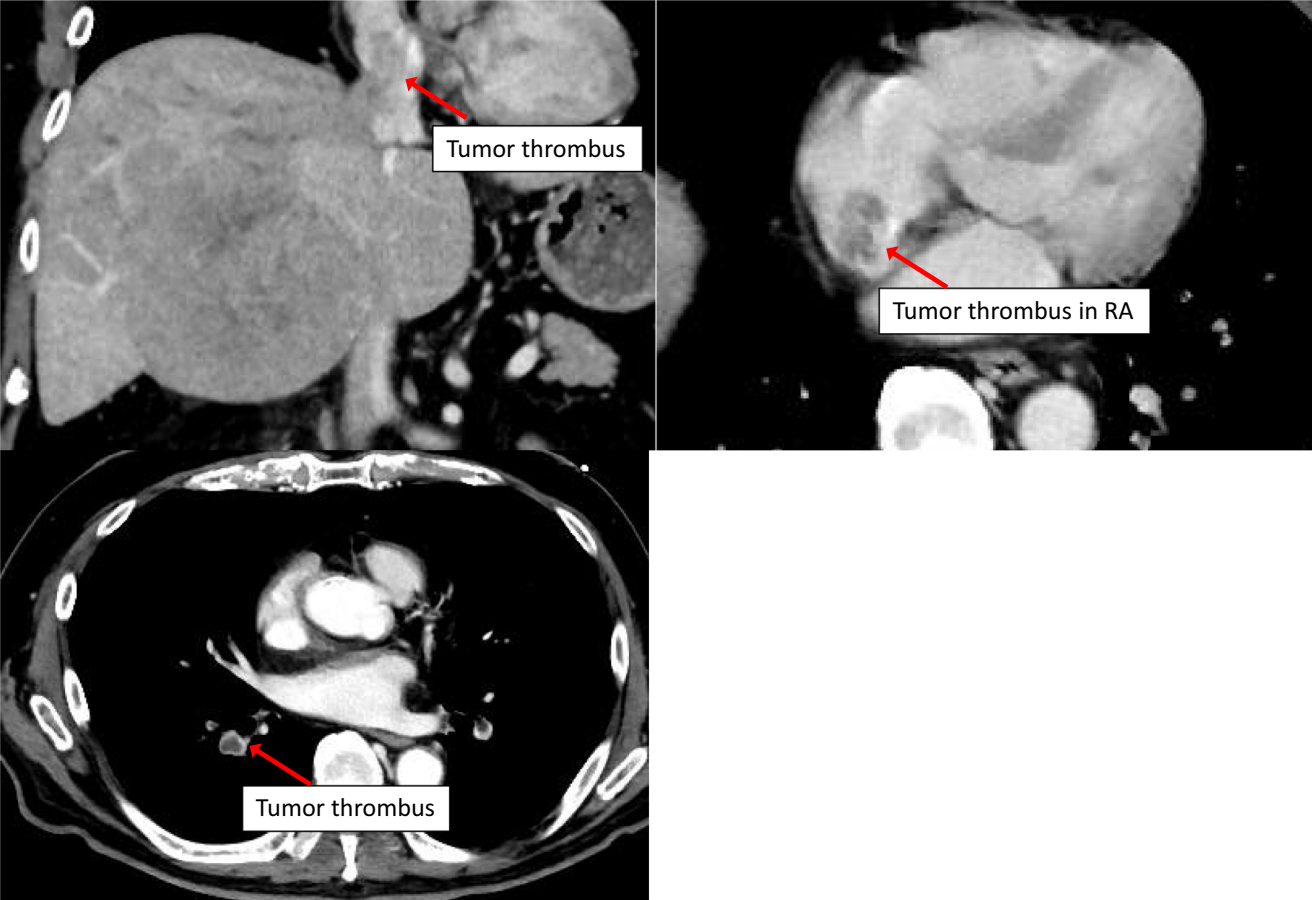
Abdominal computed tomography showed a pulmonary embolism and the tumor thrombus extension into the right atrium

**Fig. 3:**
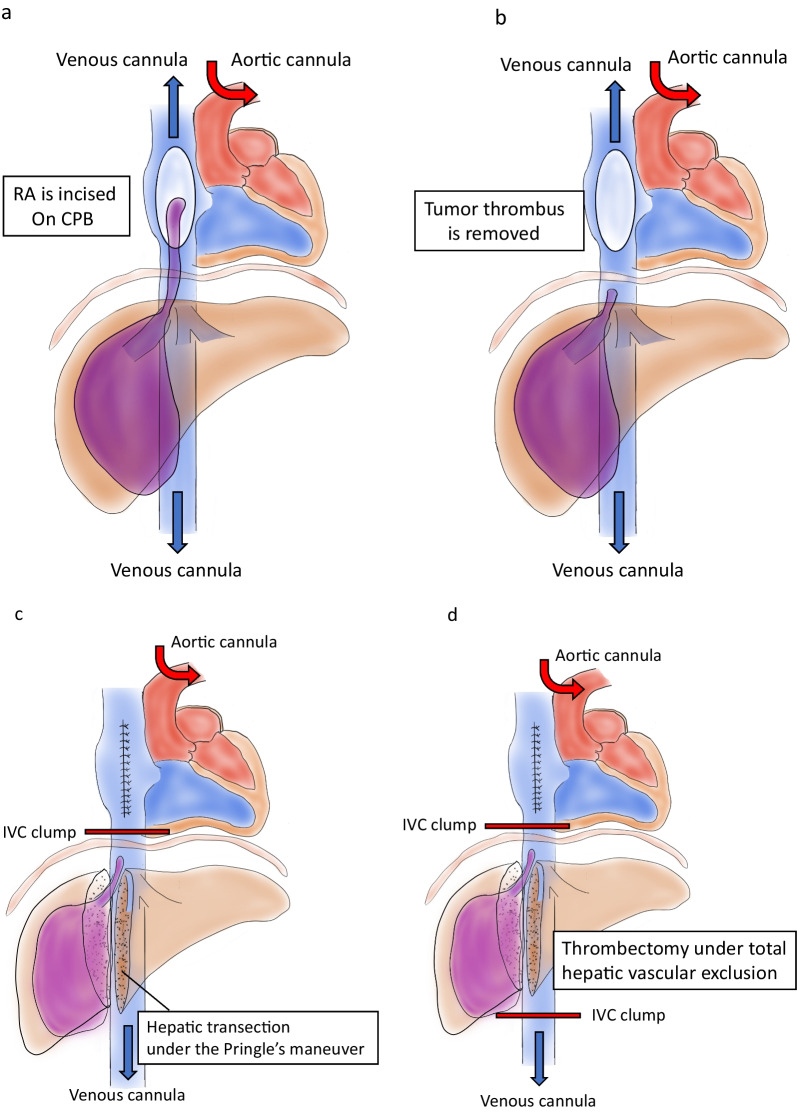
Surgical schema

A 64-year-old man was referred to our hospital for further examination of a tumor in the right lobe of the liver, which was found on computed tomography (CT) by his previous physician. He had a history of stroke and diabetes mellitus but was generally in good condition, and his liver function was rated as Child–Pugh A and Liver damage A. Abdominal CT showed a tumor on the right side of the liver with TT extending to the IVC (Fig. 1). The patient was diagnosed with HCC and scheduled for elective surgery. While awaiting surgery, he visited our hospital with complaints of respiratory distress. CT scan showed a pulmonary embolism due to TT dispersion, and the TT extension into the right atrium (Fig. 2). The TT was rapidly growing, putting the patient at a high risk of sudden death. Since the TT extended into the RA, we concluded that it would be difficult to remove it using conventional right lobectomy. After consultation with a cardiovascular surgeon, we decided to perform a right atrial incision under artificial cardiopulmonary support to remove as much of the TT as possible, followed by right lobectomy to prevent TT dispersion during operation. After opening the abdomen and confirming that there was no disseminated lesion, the cardiovascular surgeon performed a median sternotomy and cardiopulmonary bypass (CPB) was established with cannulation of the ascending aorta, superior vena cava, and inferior vena cava. The RA was incised after taping the IVC (Fig. 3a) and the TT extending from the IVC in the RA was removed as much as possible (Fig. 3b). Subsequently, the IVC was clamped to prevent TT dispersion, and resection of the right liver lobe was performed using the Pringle maneuver (Fig. 3c). After liver resection, the right hepatic vein was incised at the confluence of the inferior vena cava under total hepatic occlusion, and the TT from the right hepatic vein to the IVC and specimen were removed(Fig. 3d). The inferior vena cava (IVC) defect was sutured directly. After weaning from the CPB, vitamin K was administered to antagonize heparin, and FFP was administered to obtain hemostasis. The operation time was 12 h 47 min, CPB time was 7 h 41 min, and blood loss was 17,505 ml.

On the second postoperative day, the total bilirubin level increased to 4.5 mg/dl, but then plateaued and subsequently normalized. Postoperative complications included SSI corresponding to Clavien Dindo-II. Prophylactic antibiotic administration caused drug eruption, generalized skin rashes, and fever, which improved gradually. The patient was discharged from the hospital on postoperative day 74 because of the time required for rehabilitation due to the decline in ADL caused by these complications.

Eight months after surgery, the patient was diagnosed with hepatic and adrenal recurrence, and bevacizumab and immune checkpoint inhibitors were initiated.

He is still alive 1 year and 10 months after the surgery.

## Discussion

In previous studies, the frequency of HCC with IVC-TT was approximately 1.4% [4]. However, the treatment for HCC with IVC-TT remains controversial because of its rarity. TT extending into the RA can lead to sudden death and requires immediate surgical intervention [5, 6]. Although several reports have shown the effectiveness of surgery [3, 7], there are no definitive surgical procedures because of the rarity of the disease.

In general, hepatic resection with IVC-TT is a high-risk procedure, with a mortality rate of 15% [3, 8].

Recent improvements in the surgical techniques for total hepatic vascular exclusion (THVE), CPB, and the Cell-Saver technique have improved the safety of the procedure [3, 9–11]. When CPB is used, adverse events such as heparin-induced coagulopathy, cerebral infarction, immune disorders, and tumor dissemination may occur [11–13]. However, CPB is useful for intraoperative circulatory stabilization and is considered essential in cases of HCC with TT extending into the RA [7], as in our case. Our search of the English literature (PubMed) with descriptions of CPB time and blood loss revealed seven cases of HCC resection with TT extending into the RA under CPB [9, 10] (Table 1). Except in our case, liver resection was performed prior to thrombectomy, and the CPB time tended to be short. In our case, because the patient had a pulmonary embolus before surgery, and the risk of thrombus dispersion was considered very high, as much thrombus as possible was removed under CPB, and the liver resection with CPB was performed after clamping the IVC to prevent TT dispersion during operation. In fact, there was no tumor dispersal, and no serious complications were observed during operation and the postoperative course. However, the CPB time was prolonged, and the amount of bleeding increased due to coagulopathy caused by heparin. Extracorporeal bypass from the IVC to the SVC or RA could have been considered instead of CPB if intraoperative circulatory stabilization had been maintained [14]. However, after consulting with a cardiovascular surgeon, it was determined that extracorporeal bypass would not preserve intraoperative systemic hemodynamics. The amount of blood loss in this case may be unacceptable compared to the results of multicenter studies of HCC with TT in the RA published from Japan in recent years [15]. Although surgical techniques need to be improved to reduce blood loss, liver resection under CPB may be an option to prevent intraoperative death due to tumor dispersal.

**Table 1:** Surgical outcomes for 8 patients including the present patient and pooled data from the literature

	Wakayama et al. [9] (*n* = 6)	Ohta et al. [10]	Our report
Surgical duration (min)			
Mean ± SD	608 ± 169	739	887
Blood loss (ml)			
Mean ± SE	6540 ± 5404	5881	17,505
CPB time (min)			
Mean ± SD	32.2 ± 18.3	39	461
Hospital stay (days)			
Mean ± SD	21.2 ± 4.6	24	70

The prognosis for HCC patients with IVC or RA is very poor, with MST within 5 months in untreated patients [3, 8]. Complete resection in patients with IVC-TT is challenging; hence, molecularly targeted agents such as sorafenib could be a treatment option [16]. Recently, the efficacy of immune checkpoint inhibitors in HCC has been suggested [17]. In addition, TACE, chemotherapy, and RFA are treatment options; therefore, multidisciplinary treatment, including surgery, could improve the prognosis of patients with HCC and TT. However, surgery may also be necessary early after diagnosis to prevent sudden death from tricuspid valve closure or pulmonary embolization due to tumor dispersal.

## Conclusion

Our case demonstrates that hepatic resection with CPB is an option for HCC with TT reaching the RA. The removal of the TT before hepatectomy to prevent tumor dispersal was useful approach.

## Data Availability

Not applicable.

## Abbreviations

HCC: Hepatocellular carcinoma
TT: Tumor thrombus
IVC: Inferior vena cava
RA: Right atrium
HVTT: Hepatic vein tumor thrombus
CT: Computed tomography
CPB: Cardiopulmonary bypass
THVE: Total hepatic vascular exclusion
